# Determination of Phenolic Content, Antioxidant Activity, and Tyrosinase Inhibitory Effects of Functional Cosmetic Creams Available on the Thailand Market

**DOI:** 10.3390/plants10071383

**Published:** 2021-07-06

**Authors:** Sariya Mapoung, Warathit Semmarath, Punnida Arjsri, Sonthaya Umsumarng, Kamonwan Srisawad, Pilaiporn Thippraphan, Supachai Yodkeeree, Pornngarm Limtrakul (Dejkriengkraikul)

**Affiliations:** 1 Department of Biochemistry, Faculty of Medicine, Chiang Mai University, Chiang Mai 50200, Thailand; srmapoung@gmail.com (S.M.); warathit_semmarath@cmu.ac.th (W.S.); punnida_dream@hotmail.com (P.A.); k.srisawad@gmail.com (K.S.); tipprapant@gmail.com (P.T.); yodkeelee@hotmail.com (S.Y.); 2 Center for Research and Development of Natural Products for Health, Chiang Mai University, Chiang Mai 50200, Thailand; sonthaya.u@cmu.ac.th; 3 Department of Veterinary Biosciences and Veterinary Public Health, Division of Veterinary Preclinical Sciences, Faculty of Veterinary Medicine, Chiang Mai University, Chiang Mai 50200, Thailand

**Keywords:** whitening cream, plant phenolics, cosmetic plants, cosmetic products, hot water cream extraction, Thailand

## Abstract

Recently, the global trend toward the use of natural extracts and antioxidant agents in the cosmetic cream industry to produce whitening effects has been increasing. This has also been a persistent trend in Thailand. In this study, samples of commercial cosmetic creams on the Thai market were assessed for a functional evaluation of their antioxidant activity, tyrosinase inhibitory effects, and phenolic contents. Samples were extracted using hot water and sonication extraction method to obtain the functional cream extracts. Total phenolic contents in all samples were within the range of 0.46–47.92 mg GAE/30 g cream. Antioxidant activities of the cream extracts were within the range of 3.61–43.98 mg Trolox equivalent/30 g cream, while tyrosinase inhibition activities were within the range of 2.58–97.94% of inhibition. With regard to the relationship between the total phenolic content and the antioxidant activity of the cosmetic creams, Pearson’s correlation coefficient revealed a moderately positive relationship with an r value of 0.6108. Furthermore, the relationship between the antioxidant activity and the tyrosinase inhibitory activity of the cosmetic creams was highly positive with an r value of 0.7238. Overall, this study demonstrated that the total phenolic contents in the functional cosmetic creams could play a role in antioxidant activity and anti-tyrosinase activities. The findings indicate how the whitening and antioxidant effects of cosmetic creams could be maintained after the products have been formulated, as this concern can affect the consumer’s decision when purchasing cosmetic products.

## 1. Introduction

During the first decade of the 21st century, cosmetic product sales underwent fairly significant growth and represented 23% of the overall market share of consumer products. This growth was driven primarily by the Asian market [1,2]. Among these cosmetic products, the facial and skincare market has accounted for two thirds of total cosmetic sales. Interestingly, a particular aspect of the Asian skincare market would be the prominence of face-whitening products, as pale skin continues to be considered a beauty ideal in Asia [3,4]. Consequently, there has always been a strong demand for skin whiteners throughout the Asian continent [5,6].

Likewise, consumer demand continues to increase for the innovative uses of natural compounds to treat skin aging manifestations including wrinkles, sagging skin, skin texture changes, and hyperpigmentation [7,8,9,10]. Accordingly, cosmetic manufacturers have begun offering organic lines of products and natural solutions to treat a variety of problems and diseases associated with the skin including hyperpigmentation, sun burn, skin aging, erythema, rash, and atopic dermatitis [3,11,12,13,14]. In the cosmetic industry, many polyphenols obtained from plants and natural extracts hold promise as they have exhibited a range of beneficial effects on skin in both in vitro and in vivo studies [15,16,17]. Briefly, the ethanolic extract of *Spirulina platensis* inhibited ultraviolet B (UVB) irradiation-induced intracellular-ROS in human fibroblasts [18]. The treatment of human dermal fibroblasts with *Epilobium angustifolium* polyphenol extract resulted in a downregulation of the UV-induced release of MMP-1 and MMP-3, along with hyaluronidase gene expressions [19].

Phenolic compounds are plant secondary metabolites that constitute one of the most common and widespread groups of substances in plants [15,20,21]. The health-promoting effect of phenolic compounds is based on their antioxidant and anti-aging properties [22,23,24]. Many previous studies have reported on the phenolic-containing extracts that exhibit the photo-protective properties [25]. The phenolic compounds, resveratrol and quercetin, have been reported to exhibit the solar photoprotection by absorbing the UV radiation and inhibiting UV irradiation-induced oxidative stress and inflammation in human keratinocytes [26,27]. A protective effect of topically applied EGCG from green tea was observed against acute skin damage caused by UVA in a rat model [28]. Moreover, the algae-derived phenolic compound, dieckol, could suppresses airborne particulate matter-induced skin aging in vitro and in a zebrafish model [29]. With regard to clinical studies, formulations containing green tea extract could protect against UV-induced photoaging and photo-immunosuppression in volunteer human subjects [30]. Accordingly, plant extracts that are rich in the phenolic compounds that exhibit antioxidant and anti-skin aging properties, will have a high potential to be developed into advantageous cosmetic formulations including facial anti-aging creams and serums, dry skin hydrating gels, antioxidant-based lotions, anti-allergy skin creams, and hair care products [20,31,32,33,34].

Cosmetic creams that are high in phenolic compounds have been used to fight against environmental free radicals that are known to lead to certain skin damaging conditions [35]. These conditions can manifest as minor skin problems (heat, pain, erythema, and wrinkles) or major skin disorders (autoimmune diseases, psoriasis, and skin aging) that can potentially develop into skin cancer [20,36,37,38,39,40]. The free radicals caused by chronic exposure to sunlight or UV radiation have been proven to be a major cause of skin cancer [41,42]. With regard to the skin cancer, melanoma is the most common type of skin cancer in white-skinned populations [43]. Importantly, increasing incidences of skin cancers around the world are largely attributed to increased exposure to UV radiation [44,45]. Australia has long had the highest recorded incidence of melanoma throughout the world due to a combination of a largely white-skinned population, high ambient UV radiation levels, and the outdoor lifestyle of the country’s populous [46,47]. In 2018, Australia had 12,265 newly diagnosed cases of melanoma with the highest age-standardized incidence rate of 33.6 per 100,000 [48,49]. In Australia, it is popular to use sunless tanning skin products that contain dihydroxyacetone (DHA) as the main ingredient [50,51]. These products protect the user’s skin against UV radiation by producing melanoidins (to imitate the natural skin tan caused by melanin) which react with the protein and amino acid composition in the surface layers of the skin resulting in the change in skin color. However, most sunless tanning products do not contain sunscreen. Therefore, they do not provide the necessary protection against UV radiation [51,52].

Unlike Australia, there are cultural differences throughout Asian countries that dictate how the people choose to apply skin cosmetic products [53,54]. In contrast to Australian people, Asians, who usually prefer fair or white skin, would rather use skin cosmetic products with the whitening ingredients. These ingredients include vitamin C, its derivatives, and various plant extracts (*Glycyrrhiza glabra*, *Centella asiatica*, *Acacia confusa*, *Cassia fistula*, *Vitis vinifera*) that aim to reduce skin pigmentation [3,55,56,57,58]. These products (whitening creams, anti-aging serums, toners, lotions, facial foams, and soaps) are commonly used by Asian consumers to whiten the skin and treat yellowish discoloration or brown patches on the face or body [59,60,61,62]. In addition to whitening effects, many phenolic compounds derived from plant extracts have been reported for their anti-tyrosinase activity and are now being included in cosmetics ingredients [3,16,63]. Natural phenolic compounds, including kaempferol, caffeic acid, and resveratrol, have exhibited the anti-tyrosinase activity [33,57]. The benzofuran moracin M, which was found in *Morus alba* has shown potent tyrosinase inhibition activity [64,65]. Moreover, *Cassia fistula* and *Camellia sinensis* extracts exhibited the anti-tyrosinase activity, antioxidant activity, and anti-aging properties, and can be use as cosmetic ingredients [58,66]. With regard to the ingredients used in whitening cosmetic products, the cosmetic products that are available nowadays on the market usually contain vitamin C or its derivative forms in cosmetic formulations [67,68]. Vitamin C or L-ascorbic acid is a water-soluble vitamin that exhibits antioxidant capacity and helps to protect the body from free radical damage. Many cosmetic and pharmaceutical industries have been focused on introducing this vitamin in formulations (creams and serums) that can be applied on the skin to provide some essential properties including, skin whitening, anti-wrinkling features, and protection against UV-induced damage [67,68,69]. Therefore, vitamin C, together with these aforementioned natural extracts, is now being added to whitening cream formulations.

In order to trace and determine the stability of phenolic compounds and other essential ingredients (vitamin C, vitamin E, and anti-tyrosinase inhibitor agents) in cosmetic creams after the products have been formulated, many analytical methods, including solid-liquid extraction using ultrasound [70], hot water extraction and sonication [71], liquid-liquid extraction [72], solid phase extraction [73], microwave-assisted extraction [74], and high-performance liquid chromatography with mass spectrometry, have been developed to determine the amount of total phenolic compounds, synthetic antioxidants, or active ingredients in the cosmetic creams [70,71,75]. Cosmetic scientists are now paying specific attention to the presence of phenolic compounds (resveratrol, benzoic acid and its derivatives, cinnamic acid, ferulic acid, and EGCG) [70,76,77] in skincare products with the hopes of satisfying the cosmetic consumers with regard to the maintenance of functional cosmetic cream properties (whitening and anti-hyperpigmentation effects) in relation to what the products have claimed in their cosmeceutical research and marketing materials [23,78]. Therefore, this study aimed to determine the total phenolic contents, antioxidant activities, and tyrosinase inhibitory effects, of some of the commercial cosmetic creams that are available on the Thai market. The relationship between the phenolic contents and the relevant bioactivities detected in these cosmetic creams will also be studied. The information obtained from this study would be beneficial to consumers who purchase cosmetic products available on the Thai market. To our knowledge, this is the first report to identify and assess the total phenolic contents and their relevant bioactivities directly obtained from commercial cream products on the Thai market.

## 2. Results

### 2.1. Characteristics of the Cosmetic Creams in This Study

**Figure 1:**
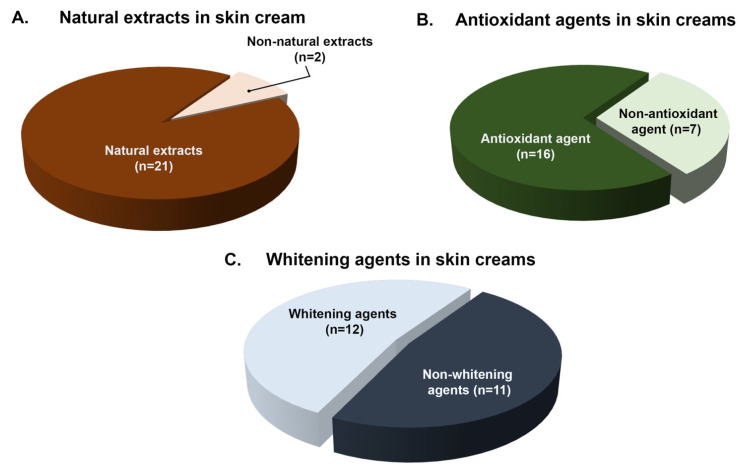
The characteristics of the studied cosmetic cream products from Thai market (Total number of samples = 23).

The presence of different ingredients in the product formulation, including plant extracts, antioxidants, and anti-tyrosinase agents were based on the cream products’ label appearing on the 23 functional cosmetic creams shown in Table 1. Figure 1 represented the descriptive data for the 23 cosmetic cream samples on the Thai market in different characteristics. Overall, among all 23 cream products, plant extracts were listed as essential ingredients and appeared in the products’ marketing materials. The plant extracts included are *Vitis vinifera*, *Centella asiatica*, *Panex ginsen*, *Oryza sativa*, *Passiflora laurifolia*, *Aloe barbadensis*, *Citrus aurantium dulcis*, *Lepidium sativum*, *Hamamelis virginiana*, and *Enantia chlorantha*. Based on the labels of all 23 cream products, 91% of the total studied creams had indicated that they contain various natural extracts in their formulations as is shown in Figure 1A. Additionally, Figure 1B showed that 16 out of 23 of the cream products had stated in the label that they contain well-known antioxidant agents including vitamin C (ascorbic acid, ascorbyl glucoside, and ascorbyl tetraisopalmitate) [67,68] and vitamin E (tocopherol and tocopheryl acetate) [69], which accounted for 70% of the total studied creams. Moreover, as appearing on the products’ labels, a variety of well-known tyrosinase inhibitor agents (kojic acid, arbutin, niacinamide, glutathione, and citric acid) [62,79,80] appeared on the label of the cream products, accounting for 52% of the total studied creams (12 out of 23) as is shown in Figure 1C.

### 2.2. Determination of Total Phenolic Contents in Cosmetic Creams

In order to prepare the cream extract, a total of 1 g of each commercial cream sample was extracted for the phenolic content determination using hot water extraction and sonication procedure. Prior to the cream extraction experiment, we did validate our method by adding an exact amount of a standard phenolic compound (5 mg of gallic acid) [81] incorporated into the cream-based emulsion, and the process of extraction was undertaken. A series of three replicated extractions of the same cream were performed on the same day, while the % of recovery was recorded. The results obtained for all of the phenolics content experiments revealed a more than 95% of recovery.

Table 1 showed the ingredients contained in each of the 23 cream samples and their respective total phenolic contents and antioxidant capacity. Among the 23 cream extracts, the quantity of the phenolic compounds ranged between 0.46–47.92 mg GAE/30 g cream (representing the amount in one cosmetic jar container) with the mean value of the total phenolic contents recorded at 9.84 ± 2.10 mg GAE/30 g cream. Interestingly, when calculating the mean for each group of the cosmetic cream samples according to the presence of natural extracts in the product’s label, the mean value of the total phenolic contents in the cream product that contained a natural extract group in the formulation was accounted for 10.14 ± 2.27 mg GAE/30 g cream; while the mean value of the total phenolic contents in the cream product that contained no natural extract in the formulation was accounted for 6.71 ± 3.30 mg GAE/30 g cream.

### 2.3. Determination of the Antioxidant Activity from Cream Extracts

**Figure 2:**
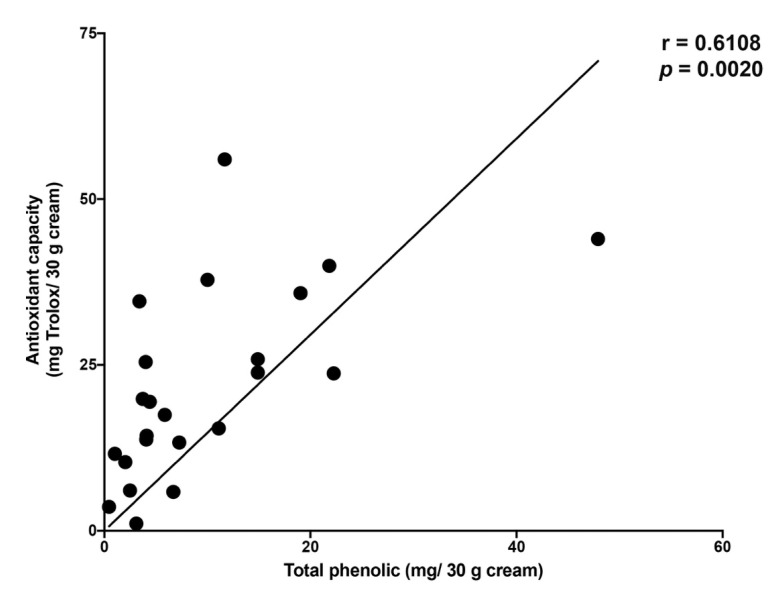
Scatter plot and Pearson’s correlation coefficient (r) for the relationship between total phenolic content and antioxidant capacity of 23 cream products on the Thai market.

In order to determine the antioxidant capacity of the cream extract, a 0.1 mL portion of the cream extract from each of the 23 cream products was used to measure the antioxidant activity by ABTS assay. The ingredients contained in each of the 23 cream samples and their respective antioxidant activity (represented as the unit of milligram of Trolox equivalent per 30 g of cream) is shown in Table 1. Based on the product marketing materials, many synthetic antioxidant agents have been added to the cream formulations including ascorbic acid, ascorbyl glucoside, ascorbyl palmitate, ascorbyl tetraisopalmitate, and sodium ascorbyl phosphate, tocopherol and tocopheryl acetate. The antioxidant properties of the 23 cream extract samples ranged between 3.61 and 43.98 mg Trolox equivalent/30 g cream (one jar container) with an average value of 21.71 ± 4.63 mg Trolox equivalent/30 g cream. When calculating the mean value for the cream samples that included antioxidant agents in the formulation, the mean for this group was accounted for 22.60 ± 5.84 mg Trolox equivalent/30 g cream; while the mean value for the group of cream samples that contained no antioxidant agents was accounted for 19.66 ± 8.03 mg Trolox equivalent/30 g cream. To establish the relationship and trendline of the cream extract samples based on two continuous variables obtained from our data (total phenolic contents and antioxidant capacity), we created the scatter plot of 23 cream samples according to the total phenolic contents (*x*-axis) and antioxidant capacity (*y*-axis) as demonstrated in Figure 2. Furthermore, we employed Pearson’s correlation analysis to confirm the relationship between the phenolic contents and antioxidant capacity among the cream samples, and the correlation coefficient (r value) was equal to 0.6108 (*p* = 0.0020), which indicated a moderate level of positive association between the phenolic contents in the cream extract and the antioxidant capacity. This finding confirmed that the quantity of the phenolic compounds found in the cosmetic products could be indicative of the antioxidant activity in the functional cosmetic creams.

### 2.4. Determination of the Tyrosinase Inhibitory Activity from Cream Extracts

**Figure 3:**
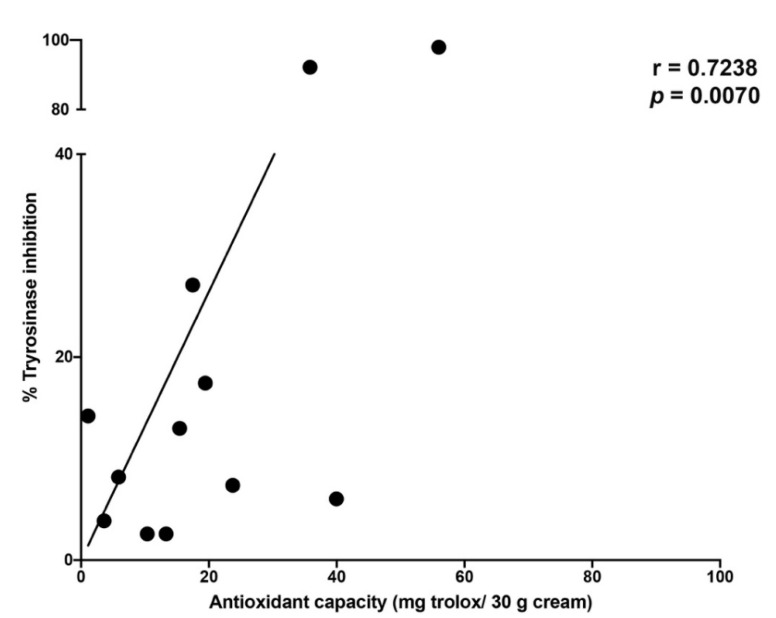
Scatter plot and Pearson’s correlation coefficient (r) of the relationship between total antioxidant capacity and % tyrosinase inhibition from 12 cream products available on the Thai market.

In addition to the antioxidant capacity of the cream extracts, the anti-pigmenting or whitening effects of these products could be traced by determining the inhibitory effect of tyrosinase activity. The cream extract from each of the 23 cream samples were determined for anti-tyrosinase activity using mushroom tyrosinase spectrophotometry assay. Many of the studied creams contained well-known whitening agents including kojic acid, arbutin, niacinamide, glutathione, citric acid [62], and some phyto-extracts (*Glycyrrhiza glabra* [56], *Morus alba* [64], *Aloe barbadensis* [82], *Oryza sativa* [83], and *Lepidium sativum* [84]. The ingredients that appeared in the product’s label in each of the 23 cream samples and their respective tyrosinase inhibitory activity is shown in Table 2 and found that, 12 out of the 23 cream extracts inhibited mushroom tyrosinase activity, accounting for 52% of the total studied cosmetic creams. The tyrosinase inhibition activity varied from 2.58 to 97.94% of inhibition with a mean value of 24.37 ± 5.20 inhibition. When calculating the anti-tyrosinase mean value for the cream samples that included whitening agents in the formulation, the mean for this group was accounted for 33.71 ± 10.16% inhibition; while the mean value for the group of cream samples that declared no whitening agents was accounted for 10.60 ± 3.35% inhibition. Kojic acid, a well-known anti-tyrosinase agent, was used as a positive control and it showed 52.92 ± 0.46% inhibition. Furthermore, we used the scatter plot to establish the trendline among the cream extract samples based on two continuous variables (tyrosinase inhibition activity and antioxidant capacity) as demonstrated in Figure 3. The relationship among the cosmetic cream samples concerning the tyrosinase inhibitory activity and their antioxidant capacity was determined using Pearson’s coefficient correlation [85]. It was found that among the 23 cosmetic creams, there was a moderately positive association between these two factors with an r value of 0.4754 (*p* = 0.0219) (data not shown). However, when the group of cosmetic cream samples with detectable tyrosinase inhibition activity were assessed for the relationship between antioxidant capacity and tyrosinase inhibition activity, a high positive relationship between these two factors was observed with an r value of 0.7238 (*p* = 0.0070). The results provide useful information with regard to the strong relationship between tyrosinase inhibitory activity and antioxidant activity. To date, no detailed reports on the tyrosinase inhibitory activity of functional cosmetic products are available.

## 3. Discussion

In many Asian countries including Thailand, over 80% of the face creams on the market claim to deliver whitening properties [4,6,86]. In this study, for commercial ethical reasons, the actual names of all 23 brands of cosmetic creams have not been disclosed. Only the active ingredients of each cosmetic cream formulation that appeared on the product’s label are provided in Table 1. A cosmetic cream is a matrix consisting of water and oil phases based on two emulsion types: water in oil or oil in water [70,87]. With regard to the suitable cream extraction method for each type of cosmetic cream in this study, the studied creams that are commonly used in Thailand are mostly comprised of oil in water and are emulsion-based in such a way that they can easily be diluted in water. Accordingly, the hot water extract followed by sonication technique was employed in this study to separate the active ingredients from the oil phase of the cream matrix and to obtain functional cream extracts. The product extracts underwent further testing for the determination of their total phenolic content, antioxidant capacity, and tyrosinase inhibitory activity. In agreement with the extraction methodology of our study, previous studies have employed the hot water extraction with sonication to determine for the total phenolic compounds in the cream samples on the Syrian market [71]. Other studies have used the water-based extraction procedures but with different extraction solvents including deionized water in solid-liquid extraction [76]. The 70% ethanol, hexane, and water have also been used as extracting agents to de-emulsify the phenolic compounds obtained from the creams and similar emulsions [70]. Another study used absolute ethanol extracting agent to determine the polyphenol compounds (tannins and flavonoids) in cosmetic creams as well [72,77].

The cosmetic industry has undertaken several measures to date to increase the stability of the active ingredients used in cosmetic products. These measures employ conjugates to change the molecular characteristics of the natural compounds, as well as to add certain synthetic antioxidants (vitamin C, vitamin E, and their derivatives) and anti-hyperpigmentation agents, including anti-tyrosinase inhibitors (kojic acid and arbutin), and whitening synthetic compounds (niacinamide, glutathione, and citric acid) [21,22,23,68,88]. The phenolic compounds in plant extracts promoted the skin whitening effects mechanistically via the removal of free radicals and the chelation of metal ions [89,90]. The strong antioxidant properties of the phenolic compounds can delay the aging process of the skin by counteracting the photo-aging process through the prevention of UV penetration, a reduction in inflammatory responses and oxidative stress, and by influencing specific survival signaling pathways (MAPK and PI3K/Akt pathways) [29,35,91,92]. Moreover, phenolic compounds exhibit depigmenting properties, by controlling the activity of tyrosinase, which is the key enzyme involved in the skin melanogenesis [3,16]. In addition to the direct suppression of tyrosinase catalytic activity, other mechanisms responsible for these depigmenting effects are included, disruption of melanogenesis via the post-transcriptional control of tyrosinase [88], the regulation of melanosome transfer, and the suppression tyrosinase transcription by suppressing the upstream MAPK signaling pathway [80,93,94]. Therefore, for these reasons, the phenolic compounds are now being actively used in the field of cosmetology and dermatology, as well as in aesthetic medicine.

The list of essential ingredients of all 23 functional cosmetic creams in Table 1 indicate the different phenolic-containing extracts that serve as the active ingredients in these cream formulations. Our data indicate that the phenolic contents varied among the 23 cosmetic cream products ranging from 0.46–47.92 mg GAE/30 g cream. The wide range of total phenolic contents found in the cosmetic cream samples were also reported in another previous study as the total phenolic contents among cosmetic cream samples in the Syrian market ranged from 2.9–29.8 mg GAE/g cream [71]. According to the product marketing materials, many plant extracts have been added to the cream formulations, and were previously reported for their high content of phenolic compounds and showed high amount of total phenolic contents in our study; these extracts were included, *Vitis vinifera* (sample No. 1) [13], *Citrus* species (sample No. 2 and No. 23) [95], *Glycyrrhiza glabra* (sample No. 18) [56], *Aloe barbadensis* (sample No. 10, No. 12, and No. 14) [96], and *Schizandra chinenisi* and *Scutellaria baicalensis* (sample No. 20) [97,98]. Nevertheless, some of the previous high phenolic-containing plant extracts that were included in the formulation of the cream samples showed very low or little amount of total phenolic content when tested; these extracts were included, *Morus alba* (sample No. 15) [64], *Morus nigra* (sample No. 5) [99], and *Oryza* *sativa* (sample No. 16, No. 17, and No. 19) [83]. Interestingly, the contradicted amount of total phenolic contents were also observed in the same plant extracts that were included in different cream samples; these extracts were *Hamamelis virginiana* (high amount of total phenolic content in sample No. 14, but low in sample No. 7) [100], *Solanum lycopersicum* (high amount in sample No. 23, but low in sample No. 7) [101], and *Centella asiatica* (high amount in sample No. 10, but low in sample No. 5) [55]. The reason might be due to a different quantity of the certain extract added to the cream formulation in each cream sample. Unfortunately, the percentage or exact amount of the extracts were not included in the product’s label of the 23 cream samples, which complied with product registration requirements.

In addition to the aforementioned plant extracts, some of these products contained synthetic antioxidant compounds in their formulations. Vitamin E was commonly identified in many of these products for displaying antioxidant effects [69]. Other products contained numerous plant extracts with the addition of synthetic antioxidant compounds, while some products contained only plant extracts without the addition of any antioxidant compounds. Vitamin C is well-known for its strong antioxidant and free-radical scavenging properties [67,68,102]. Thus, it is not surprising that this compound, or its derivatives, was one of the active ingredients contained in many of the functional cosmetic creams. In our study, among the 23 cosmetic creams, 50% of the studied creams indicated that they contained vitamin C on the list of active ingredients of the product, while another 50% of the studied creams stated that they contained plant extracts that could promote a skin whitening effect. Based on labels of each of the 23 cream samples, it might be difficult to establish a relationship between the phenolic contents and the antioxidant activity of the cosmetic creams because of the presence of different types of plant extracts included in each cream formulation. Herein, our study demonstrated a moderately positive relationship between the phenolic contents (mg GAE/30 g cream) and the antioxidant capacity (mg of Trolox equivalent) among the studied creams on the Thai market (r value = 0.6108). The findings of our study are in agreement with a previous study that investigated 11 plant species of *Sargassum* extracts. A positive correlation between the phenolic contents and their antioxidant activity was significantly observed [63].

In addition to the antioxidant effects, another biological benefit of the studied creams was a declaration of a skin whitening effect. As a variety of anti-tyrosinase and anti-hyperpigmentation agents have been listed on the labels of the 23 cream samples, their mechanisms for skin whitening and anti-hyperpigmentation are diverse. Arbutin and kojic acid can directly inhibit the tyrosinase activity via the act of a competitive enzyme inhibitor [16,88], while other whitening agents, such as niacinamide, works by inhibiting melanosome transfer from melanocytes to keratinocytes’ skin cells [80]. Glutathione acts both directly via the inhibitory binding at the copper-containing active site of the tyrosinase enzyme and by quenching the free radicals that contribute to tyrosinase activation and melanin formation [93]. Moreover, citric acid acts as the upstream gene regulators for the melanogenesis and tyrosinase activity [94].

According to the product marketing materials, many natural extracts and anti-tyrosinase agents have been added to the cream formulations and these were previously reported for their tyrosinase inhibition activity and showed the potent anti-tyrosinase activity in our study; these extracts were included, kojic acid (sample No. 14) [62], jelly fish extract (sample No. 22) [103], citric acid (sample No. 2, 12, and 18) [94], and ascorbyl palmitate (sample No. 23) [54,104]. Nevertheless, arbutin (sample No. 7, 17 and 20) [62], a potent anti-tyrosinase agent, showed very low or non-detectable levels of anti-tyrosinase activity when tested. Interestingly, the contradicted anti-tyrosinase activity was observed in the same plant extracts or anti-tyrosinase agents that were included in different cream samples; these extracts were *Aloe barbadensis* (high in sample No. 12 and 14, but low in sample No. 3, 4 and 10) [82], *Lepidium sativum* (high in sample No. 12 but not detectable in sample No. 13) [84], *Morus alba* (high in sample No. 15, but low in sample No. 4) [65], and *Oryza sativa* (high in Sample No. 15 and 16, but low in sample No. 17 and 19) [83]. The reason might be the same issue as the total phenolic contents as the different quantity of the certain extract was added to the cream formulation in each cream sample. Moreover, a plant extract could exhibit synergistic or antagonistic effect when used in conjunction with another plant extract, in which the effects of active ingredients are concealed by other compounds in a complex mixture in the cream product formulation [105,106,107,108].

In this study, among the 23 cream products, it was difficult to establish the relationship between phytochemicals and the tyrosinase inhibitory activity as the r value exhibited a weak relationship. Similarly, previous study of a hundred extracts obtained from plants collected in India, Africa, and the Mediterranean area also exhibited a weak correlation between total phenolic content and anti-tyrosinase activity (r = 0.3535) [16]. Nevertheless, it was found that there was a high positive relationship between the antioxidant and tyrosinase inhibitory activity among the 23 cream samples with an r value of 0.7238. Accordingly, a profoundly positive relationship was observed between total phenolic contents and antioxidant activity, as well as between the antioxidant activity and anti-tyrosinase activity, among the cream samples on the Thai market. It can be assumed that these biological properties in these functional cosmetic creams could potentially be derived from the inclusion of phenolic-containing plant ingredients and the extracts in the cosmetic creams. Nevertheless, it is important to determine the exact amount of active ingredients presented on the label of each cosmetic cream product. Either the use of metabolic profiling based on gas chromatography of the pure compounds, or the verifiable analytical methodology based on chromatography with mass spectrometry of the active compounds’ detection would accomplish more detailed information of the ingredients’ mixture of the plant extracts in cream products and their synergistic or antagonistic bioactivities [105].

## 4. Conclusions

Overall, this study revealed important information regarding the total phenolic contents that could be indicative of the skin whitening and antioxidant effects of the studied products. The findings of this study could also shed light on how these products can maintain their stability by detecting the phenolic contents after a product has been formulated as well as their bioactivities. In order to gain the consumer’s trust with regard to the effectiveness of the antioxidant and tyrosinase activity of cosmetic products, further determinations on the exact amounts of the active ingredients would confirm whether those ingredients were actually included in the cream formulations as has been claimed on the products’ labels.

This study would be beneficial to cosmetic manufacturing companies that are tracing the cosmetic product’s effectiveness and to ensure the stability of the phenolic compounds and the biological activities in the cosmetic products. As nowadays, many cosmetic cream brands have made claims with regard to the whitening and antioxidant effects of their products, the critical information obtained from this study would be beneficial to consumers when deciding whether to purchase a cosmetic product available in the Thai marketplace. On the other hand, this report could encourage manufacturers to improve the quality control standards of their skincare products, either before or after the product formulation process, as well as the claims they make with regard to the bioactivities described in the products’ marketing information of the given product.

## 5. Materials and Methods

### 5.1. Chemicals and Reagents

Mushroom tyrosinase, L-tyrosine to 3,4-dihydroxyphenaline (L-DOPA), a substrate for tyrosinase, and kojic acid (a positive control) were used for determining the anti-tyrosinase activity. The 2,2′-azino-bis (ethylbenzthiazoline-6-sulfonic acid (ABTS), a radical agent and Trolox reagent (a positive control) were used for determining the antioxidant capacity in ABTS assay. Folin and Ciocalteu′s phenol reagent and gallic acid (a standard phenolic compound) were used for determining the total phenolic contents in Folin–Ciocalteu as-say. All reagents above were purchased from Sigma-Aldrich (St. Louis, MO, USA).

### 5.2. Samples Used in This Study

Cosmetic creams were purchased from supermarkets or department stores located in the northern part of Thailand, all of which claimed to provide whitening effects. This study involved 23 functional cosmetic cream samples that were manufactured in different countries including Thailand, Korea, and Japan. The selected cream samples were based on well-known brands in the Thai cosmetic market and were approved by Thai Food and Drug Administration (Thai’s FDA). All 23 cosmetic cream products had been formulated with various plant natural extracts, synthetic antioxidant agents, and whitening agents as declared on the product’s label of individual cream samples. The full detail of the ingredients on a product’s label was shown in Table 1.

### 5.3. Preparation of Cream Extract

The extraction procedure of the cream samples was modified from previously described protocol [71]. Briefly, a total amount of 1 g of each commercial cream sample was mixed with 50 mL of freshly boiled water and mixed for 3 min. Then, the cream extract was sonicated for 10 min and put on ice for 5 min. The mixture was then centrifuged at 4000× *g* for 20 min. The suspension was kept and was further assessed for the determination of total phenolic contents, ABTS assay, and tyrosinase inhibitory activity.

### 5.4. Determination of Total Phenolic Contents

Total phenolic content in the cream extracts was determined by using the modified Folin–Ciocalteu assay, as has been previously described [58]. Briefly, 0.4 mL of each dilution of the cream extracts was mixed with 0.3 mL of the Folin–Ciocalteau reagent and kept in the dark at room temperature for 3 min. After that, 0.3 mL of carbonated sodium was added to the mixture. The mixture was then further incubated in the dark at room temperature for 30 min. The absorbance of the blue complex was evaluated at 765 nm using a UV-visible spectrophotometer and then compared to a standard curve that had been prepared with various concentrations of gallic acid (GA) solution. The total phenolic content was shown as milligrams of GA equivalents per 30 g cream (mg GAE/30 g cream).

### 5.5. ABTS Assay for Antioxidant Properties of Cream Extracts

The antioxidant activity of the cream extracts was confirmed using the ABTS assay as has been previously described [109]. ABTS radical cation was prepared by mixing 7 mM ABTS stock solution with 2.45 mM potassium persulfate (K_2_S_2_O_8_) (1/1, *v*/*v*). The mixture was incubated in the dark for 12–16 h until the reaction was completed. The assay was conducted on 990 μL of ABTS solution and 10 μL of each dilution of the cream extract. After 6 min, the absorbance was immediately recorded at 734 nm using a spectrophotometer and then compared to the standard curve which had been prepared with various concentrations of Trolox solution. The antioxidant capacity was shown as milligrams of Trolox equivalents per 30 g cream (mg Trolox/30 g cream).

### 5.6. In Vitro Tyrosinase Inhibitory Activity of Cream Extracts

Tyrosinase inhibition assay was determined following a method previously described [58]. Briefly, an aliquot (10 μL) of the cream extract at the concentration of 20 mg/mL were mixed with mushroom tyrosinase at a concentration of 100 U/mL in sodium buffer (pH 6.4) and were then added to a 96-well-plate. The kojic acid at the concentration of 5 μg/mL were used as positive control. Then, 1 mM of L-DOPA as a substrate was added into the reaction mixture. It was then incubated for 10 min at room temperature. The change of absorbance at 490 nm was measured every 1.5 min for 15 min using a microplate reader. The percent *inhibition* of *Tyrosinase* was then calculated by using the following formula:

$$Tyrosinase\;inhibition\left(\%\right)=[\frac{\left(A-B\right)}{A}]\times 100$$

*A* = Slope of vehicle control (deionized water) at 490 nm.

*B* = Slope of sample at 490 nm.

### 5.7. Statistical Analysis

All data are presented as mean ± standard error (S.E.) values. The Pearson’s coefficient correlation test was conducted via Prism version 8.0 software, and the significance was determined at * *p* < 0.05, ** *p* < 0.01 and *** *p* < 0.001 vs. the control of each experiment.

## Figures and Tables

**Table 1:** Functional cosmetic cream samples obtained from the Thai market with their respective phenolic contents and antioxidant capacities (total number of samples = 23).

Functional Cream (Individual Sample Code)	Important Active Ingredients (Declared in the Product’s Label)	Total Phenolic Contents (mg GAE/30 g Cream)	Antioxidant Capacity (mg Trolox Equivalent/30 g Cream)
No. 1	*Vitis vinifera*, Ascorbyl glucoside and Tocopherol	47.92 ± 3.94	43.98 ± 7.48
No. 2	*Citrus limon*, Ascorbyl glucoside and Citric acid	19.04 ± 1.06	35.83 ± 10.95
No. 3	*Prunus armeniaca*, *Simmondsia chinensis, Aloe barbadensis,* Ascorbyl glucoside, Tocopheryl acetate and Niacinamide	0.46 ± 0.23	3.61 ± 0.02
No. 4	*Limonia acidissima*, *Hibiscus sabdariffa*, *Morus alba*, *Aloe barbadensis*, *Cucumis sativus*, *Helianthus annuus*, Ascobic acid and Tocopherol	2.03 ± 0.95	10.35 ± 0.92
No. 5	*Morus nigra*, *Rubus idaeus*, *Prunus avium*, *Lavendula stoechas*, *Centella asiatica*, *Panex ginseng*, *Zingiber officinale*, *Buddleja officinalis*, *Fragaria vesca* and Tocopheryl acetate	1.02 ± 0.03	11.61 ± 2.76
No. 6	*Daucus carota sativa*, Sodium ascorbyl phosphate and Tocopheryl acetate	4.10 ± 1.68	14.33 ± 6.58
No. 7	*Solanum lycopersicum*, *Hamamelis virginiana*, Ascorbyl palmitate, Tocopherol, Arbutin, Glutathione and Niacinamide	4.07 ± 1.41	13.76 ± 6.60
No. 8	*Passiflora laurifolia* and Caprylyl 2-glyceryl ascorbate	3.74 ± 0.70	19.87 ± 0.48
No. 9	Ascorbyl glucoside and Tocopheryl acetate	3.40 ± 0.27	34.59 ± 1.41
No. 10	*Centella asiatica*, *Allum cepa bulb*, *Aloe barbadensis* and *Simmondsia chinensis*	14.89 ± 2.92	23.86 ± 6.09
No. 11	*Leontopodium alpinum*, *Opuntia streptacantha*, *Brassica napus* and *Cynara scolymus*	3.10 ± 0.26	1.09 ± 1.54
No. 12	*Thymus serpyllum*, *Aloe barbadensis*, *Lepidium sativum* L., *Cynara scolymus*, *Borago officinalis*, Ascorbyl tetraisopalmitate, Tocopheryl acetate, Niacinamide and citric acid	11.68 ± 3.44	55.98 ± 10.29
No. 13	*Lepidium sativum* L.	4.01 ± 0.25	25.44 ± 0.35
No. 14	*Aloe barbadensis*, *Hamamelis virginiana*, *Artemia salina*, *Helianthus annuus*, *Enantia chlorantha*, *Epilobium fleischeri, Ribes nigrum*, *Pinus pinaster*, *Cardiospermum halicacabum*, *Chlorella vulgaris, Rosmarinus officinalis*, Tocopherol, Tocopheryl acetate, Kojic dipalmitate and Niacinamide	11.10 ± 0.29	15.43 ± 3.13
No. 15	*Carthamus tinctorius*, *Morus alba*, *Peaonia suffruticosa*, *Thuja orientalis*, *Prunus persica*, *Ganoderma lucidum*, *Lilium tigrinum*, *Oryza sativa*, *Helianthus annuus*, *Lavandula angustifolia*, *Illicium verum*, *Scutellaria baicalensis* and Tocopherol	4.40 ± 2.50	19.44 ± 6.10
No. 16	*Oryza sativa* and Tocopherol	6.70 ± 1.44	5.86 ± 0.81
No. 17	*Oryza sativa*, *Saxifraga stolonifera*, *Paeonia suffruticosa, Scutellaria baicalensis*, Citric acid, Arbutin, Glutathione and Niacinamide	2.49 ± 0.65	6.08 ± 2.32
No. 18	*Glycyrrhiza glabra*, *Myrciaria dubia*, Ascorbic acid, Sodium ascorbyl phosphate and Citric acid	21.84 ± 0.52	39.95 ± 13.30
No. 19	*Dioscorea villossa*, *Oryza sativa*, *Dunaliella salina*, *Crocus chrysanthus buld, Sativum Sprout*, Tocopheryl acetate and Niacinamide	7.27 ± 9.67	13.31 ± 1.43
No. 20	*Schizandra chinenisi*, *Lilium candidum*, *Saxifraga sarmentosa*, *Raeonia suffrutocosa*, *Scutellaria baicalensis*, Glutathione, Arbutin and Citric acid	14.89 ± 0.64	25.86 ± 10.98
No. 21	Niacinamide and Glutathione	10.01 ± 1.23	37.84 ± 10.39
No. 22	Jellyfish extract and Niacinamide	5.87 ± 1.83	17.48 ± 2.32
No. 23	*Myrciaria dubia*, *Citrus aurantium dulcis*, *Solanum lycopersicum*, *Arctostaphylos uva-ursi*, Ascorbyl palmitate, Tocopheryl acetate, Arbutin, Glutathione and Niacinamide	22.26 ± 0.19	23.73 ± 5.28

**Table 2:** Functional cosmetic cream samples obtained from the Thai market with their respective antioxidant capacity and % tyrosinase inhibition (total number of samples = 23).

Functional Cosmetic Cream (Individual Sample Code)	Antioxidant Capacity (mg Trolox/30 g Cream)	% Tyrosinase Inhibition
No. 1	43.98 ± 7.48	ND
No. 2	35.83 ± 10.95	91.84 ± 0.50
No. 3	3.61 ± 0.02	3.66 ± 0.11
No. 4	10.35 ± 0.92	2.58 ± 0.81
No. 5	11.61 ± 2.76	ND
No. 6	14.33 ± 6.58	ND
No. 7	13.76 ± 6.60	ND
No. 8	19.87 ± 0.48	ND
No. 9	34.59 ± 1.41	ND
No. 10	23.86 ± 6.09	ND
No. 11	1.09 ± 1.54	14.20 ± 0.23
No. 12	55.98 ± 10.29	97.37 ± 0.81
No. 13	25.44 ± 0.35	ND
No. 14	15.43 ± 3.13	12.97 ± 1.03
No. 15	19.44 ± 6.10	17.42 ± 1.88
No. 16	5.86 ± 0.81	8.20 ± 0.49
No. 17	6.08 ± 2.32	ND
No. 18	39.95 ± 13.30	6.01 ± 1.50
No. 19	13.31 ± 1.43	2.59 ± 1.43
No. 20	25.86 ± 10.98	ND
No. 21	37.84 ± 10.39	ND
No. 22	17.48 ± 2.32	27.11 ± 1.73
No. 23	23.73 ± 5.28	7.37 ± 1.77

ND = Tyrosinase activity not detectable.
